# Long-Term Exposure to Ozone Increases Neurological Disability after Stroke: Findings from a Nationwide Longitudinal Study in China

**DOI:** 10.3390/biology11081216

**Published:** 2022-08-13

**Authors:** Jiajianghui Li, Hong Lu, Man Cao, Mingkun Tong, Ruohan Wang, Xinyue Yang, Hengyi Liu, Qingyang Xiao, Baohua Chao, Yuanli Liu, Tao Xue, Tianjia Guan

**Affiliations:** 1 Institute of Reproductive and Child Health/National Health Commission Key Laboratory of Reproductive Health and Department of Epidemiology and Biostatistics, School of Public Health, Peking University, Beijing 100191, China; 2 School of Health Policy and Management, Chinese Academy of Medical Sciences & Peking Union Medical College, Beijing 100730, China; 3 College of Environmental Sciences and Engineering, Peking University, Beijing 100871, China; 4 State Key Joint Laboratory of Environmental Simulation and Pollution Control, School of Environment, Tsinghua University, Beijing 100084, China; 5 The General Office of Stroke Prevention Project Committee, National Health Commission of the People’s Republic of China, Beijing 100053, China

**Keywords:** stroke, disability, ozone, longitudinal study

## Abstract

**Simple Summary:**

In China, ozone is a major air pollutant that has been linked to stroke incidence and mortality. However, how long-term exposure to ozone affects the life quality among stroke survivors is unknown. This study presents a longitudinal analysis of nationwide data of Chinese adults, and shows that exposure to ozone can increase the risk of post-stroke disability. Taking ambient O_3_ under control can delay the progression of neurological disability among stroke survivors.

**Abstract:**

Exposure to ozone (O_3_) is associated with stroke incidence and mortality. However, whether long-term exposure to O_3_ is associated with post-stroke neurological disability remains unknown. This study investigated the relationship based on the longitudinal analysis of China National Stroke Screening Survey (CNSSS), which included 65,778 records of stroke patients. All of the analyzed patients were followed-up at least twice. Stroke disability was assessed using the modified Rankin scale (mRS). Long-term exposure was assessed by the peak-season or annual mean of maximum 8-h O_3_ concentrations for 365 days before the mRS measurement. We used fixed-effect models to evaluate the associations between O_3_ and mRS score, with adjustment for multiple confounders, and found a 10 µg/m^3^ increase in peak-season O_3_ concentration was associated with a 0.0186 (95% confidence interval [CI] 0.0115–0.0256) increment in the mRS score. The association was robust in various subpopulations. For secondary outcomes, for each 10 µg/m^3^ increment in peak-season O_3_, the odds ratio of an increased mRS score (vs. unchanged or decreased mRS score) increased by 23% (95% CI 9–37%). A nonlinear analysis showed a sublinear association between O_3_ exposure and risk for post-stroke disability. A saturation effect was observed at an O_3_ concentration of more than ~120 μg/m^3^. Our study adds to evidence that long-term exposure to O_3_ increases the risk of neurological disability after stroke.

## 1. Introduction

Stroke is recognized as one of the biggest causes of disability worldwide, yet its neurological burden has been well under-recognized as it was classified as a cardiovascular disease before the release of latest revision of the WHO International Classification of Disease (ICD-11) [1]. It is of significant public health interest to identify the risk factors for stroke-related disability. Among the modifiable risk factors, air pollution is the third leading contributor to the global stroke burden, accounting for 29.2% of the full burden [2]. Stroke accounts for an estimated 113 million disability-adjusted life-years (DALYs), which integrates healthy life years lost due to both premature mortality and living with disability [3]. It is estimated that stroke accounts for 2.07% (95% CI, 1.6–2.52%) of total years lived with disability (YLDs) and 5.65% of total DALYs (95% CI, 5.14–6.17%) according to the Global Burden of Disease (GBD) study in 2019 [4]. However, studies on risk factors have focused on their effects on the incidence or the years of life loss (YLLs) due to stroke, ignoring YLDs.

Increased concentrations of air pollution are strongly associated with risk for stroke [5,6,7]. Exposure to ozone (O_3_), a major air pollutant, also contributes to the risk for many neurological diseases, such as stroke, Parkinson’s disease, dementia, and multiple sclerosis [5,8]. A meta-analysis showed a weak association between short-term O_3_ and admission to hospital for stroke or mortality from stroke, with a pooled relative risk of 1.001 (95% CI, 1.000–1.002) per 10 ppb [9]. Although those studies were conducted in different regions, most evaluated the effects of short-term exposure to O_3_ on stroke. Studies investigating the relationship between short-term O_3_ exposure and stroke may have some inherited shortcomings. Ground-surface O_3_ is produced by photochemical chemical reactions between primary air pollutants (e.g., nitrogen dioxide and volatile organic compounds), and thus its short-term variations are affected by climate conditions, such as temperature. Therefore, climate variables, which directly affect human health in a complex nonlinear pattern, can be confounders for the short-term effects of O_3_. Long-term exposure to O_3_ is less affected by short-term fluctuations in climate, and its health effects can be stable. However, the relevant epidemiological evidence is sparse. The World Health Organization released the revised Global Air Quality Guidelines, which provide a recommended long-term, peak-season (i.e., maximum of 6-month moving averages) O_3_ level of <60 µg/m^3^ based on all non-accidental mortality and respiratory mortality studies [10], suggesting that the effects of O_3_ have been underestimated before. It is of public health importance to evaluate the long-term effect of O_3_, particularly in an aging society.

Compared with its incidence and mortality, disability caused by stroke and long-term prognosis of stroke, which may affect the quality of life, have been investigated less intensively. A cross-sectional study conducted in Texas from 2000 to 2012 reported that each 10 μg/m^3^ increment in daily exposure to O_3_ was significantly associated with a 0.29 (95% CI 0.06–0.51) increment of initial stroke severity (assessed by NIH Stroke Scale, NIHSS) [11]. A longitudinal study involving older Chinese adults found that each 10 μg/m^3^ increase in annual mean O_3_ exposure was associated with a 10.4% increased risk for cognitive impairment, as assessed by the Mini-Mental State Examination [12]. A California study showed that increment in O_3_ was negatively associated with verbal fluency and executive function [13]. Studies in China on the association between O_3_ and neurological disability after stroke have been challenging for two reasons: First, there are few studies that contain quantitative assessment of neurological function after stroke. Second, regulatory measurements of O_3_ have been based on monitoring stations and were not available for the full coverage of O_3_, hampering the assessment of population-level long-term exposure. The Modified Rankin Scale (mRS) assesses disability in patients who have suffered a stroke and is compared over time to check for recovery and degree of continued disability or dependence in daily activities. Compared with other stroke scales, mRS is easy to operate, can be scored by simple inquiry, and has good reliability and authenticity [14]. In this study, we used a fixed-effect model to evaluate the association between O_3_ and mRS score based on a national stroke survey in China. Our analysis included 65,778 records of stroke patients. The participants were followed up at least twice with valid mRS measurements. The potential effects of modifiers of the relationship between O_3_ and mRS score were also examined.

## 2. Methods

### 2.1. Exposure Assessment

We obtained the 10 × 10 km maximum daily 8-h averaged O_3_ concentrations in China from 1 January 2013 to 31 December 2019, from the Tracking Air Pollution in China Database (TAP; http://www.tapdata.org.cn; accessed on 9 June 2021). This database uses a data-fusion algorithm for O_3_ estimation that combines in situ observations, satellite remote-sensing measurements, and results from the community multiscale air quality model. Details of the database and the accuracy of the model can be found elsewhere [4]. By linking the community address (longitude and latitude) of each participant derived from the China National Stroke Screening Survey (CNSSS) to the nearest O_3_ grid, we matched each participant to the 10 × 10 km grid. We calculated the peak-season or the annual mean concentration during the year preceding the measurement of the mRS as long-term exposure to O_3_. Therefore, this study only included the mRS records measured from 1 January 2014, due to the limited availability of the exposure data. We also calculated O_3_ exposure using different time windows to test whether the exposure time window affected the estimated effect of O_3_.

### 2.2. Population Selection

The study population was obtained from CNSSS, an ongoing community-based stroke surveillance program in mainland China that started in 2013 [15]. The design, methods, and participants of the CNSSS program are available elsewhere [15,16,17]. Briefly, a two-stage stratified cluster sampling method was adopted for each screening. The participants were interviewed using a standardized face-to-face questionnaire to collect information on their demographic characteristics, socioeconomic status, stroke history, and risk factors for stroke by neurologists or physicians from community hospitals. The data can be obtained from the Bigdata Observatory Platform for Stroke of China (BOSC; https://www.chinasdc.cn/; accessed on 10 June 2021) [18]. Because the prevalence of stroke is relatively low among younger adults [19], CNSSS only screens residents aged 40 years and older in each community. The inclusion criteria were patients diagnosed with stroke, stroke onset preceding the mRS measurement, and at least two mRS measurements [20]. We did not exclude the patients with stroke recurrences. Recurred stroke might be a pathway to explain why O_3_ increased the mRS score among the survivors, and air pollution has been evidenced to be a risk factor of stroke incidences and recurrences. Therefore, the patients with a history of recurrent stroke might be more susceptible to air pollution. Excluding them could lead to an underestimated association. Additionally, the recurrent stroke patients might have a high probability of death, and thus tended to be ignored by our study. We introduced a method of inverse-probability weights to adjust for the missingness, as mentioned in the statistical analyses section.

### 2.3. Outcome

The primary outcome was the change in mRS score. The mRS score (range 0–6) was used to measure the degree of disability or dependence in daily activities of patients with stroke: the higher the score, the greater the disability. A score of 0 means no symptoms; 1 indicates no significant disability despite symptoms; and scores of 2–4 mean slight, moderate, moderately severe, and severe disability, respectively. A score of 6 indicates death and thus didn’t appear in our surveys on stroke survivors. The secondary outcome was the change in mRS score transformed into a dichotomous variable using cut-off values of >0, >1, >2, >3, >4, or >5.

### 2.4. Covariates

Personal information of the participants was collected by questionnaires. Participants’ demographic characteristics were documented, including age (≤45, 46–55, 56–65, 66–75, 76–85, of >86 years), sex (male or female), and region (southwest, south, northwest, northeast, north, east, or central China). Data on lifestyle-related factors were collected, including smoking status (yes or no), alcohol consumption (yes or no), exercise (yes or no; no enough exercise: frequency <3 times/week and <30 min/time or less), BMI (underweight, <18.5; normal, 18.5–24; overweight, 24–28; obese, >28), and drinking milk (yes or no; no enough milk intake: drinking <200 mL/day milk and <5 days/week or less) [21,22]. As both O_3_ concentration and status of neurological health can be seasonally varied, an indicator of season was also created based on the date of mRS measurement. In addition, a history of hypertension (yes or no), diabetes (yes or no), lipid disorders (yes or no), and atrial fibrillation (yes or no), as well as years after stroke (<1, 1, 2, 3, 4, 5–10, 10–20, or >20 years), were included as confounding factors. We used multiple imputation for the missing covariate values.

### 2.5. Statistical Analyses

Linear fixed-effect models were used to examine the associations between the change in mRS score and the ambient exposure to O_3_ with participant-specific intercepts. In the main analysis, we used the peak-season or the annual mean of O_3_ during the year preceding the mRS measurement (defined as the lag-1 year exposure) to assess the association. The linear fixed-effect models can be specified as follows:

mRS*_i_*_,*j*_~*β*_1_O_3,*i*,*j*_ + *β_2_**x**_i_*_,*j*_ + η(*i*),

where *i* and *j* denote the indexes for subject and visit, respectively; mRS*_i_*_,*j*_ denotes the diagnosed score of *i*th subject at the *j*th visit; ***x****_i_*_,_ denotes the adjusted covariates; η(*i*) denotes the fixed-effect term to characterize the participant-specific baseline risk of neurological disability; and *βs* denote the regression coefficients. The association was quantified as the change in mRS score for each 10 μg/m^3^ increment in O_3_. We also modeled dichotomous variables (as described above) as the secondary outcomes, using fixed-effect logistic regressions. We also created an additional dichotomous outcome to indicate mRS change (1: increased mRS; 0: unchanged or decreased mRS). We calculated the odds ratio (OR) to assess the association between the binary indicator of stroke disability and O_3_ concentrations.

The basic model (Model 1) only included O_3_ exposure and the progress in mRS score with time as the only covariate, and the term was parametrized as the interaction term between the temporal index and baseline age or years after stroke. We also conducted several sensitivity analyses to evaluate the robustness of the model estimates. First, we conducted four additional models, which sequentially included a series of covariates. On the basis of Model 1, Model 2 additionally included season; Model 3 encompassed several lifestyle-related covariates (i.e., smoking, drinking, exercise, BMI, and drinking milk); Model 4 included annual average PM_2.5_ as an additional covariate; and Model 5 further adjusted for hypertension, diabetes, and lipid disorders. Model 5 was considered as the full model. Because the subjects might not be randomly distributed among baseline mRS levels (a higher baseline mRS score might be associated with an increased risk for death, which makes follow-up less likely), the inverse probability weight (IPW) method was used to obtain representative estimates. Second, we explored the variation in the association between O_3_ and mRS score by stratifying the patients by region, age, years after stroke, sex, hypertension, diabetes, dyslipidemia, atrial fibrillation, drinking, smoking, physical inactivity, and BMI. Third, we evaluated the cumulative effect of different time windows of O_3_ exposure (lag of 1, 2, or 3 years) on the mRS score. A nonlinear model was applied to analyze the exposure–response association between O_3_ concentration and mRS score using smooth spline functions.

Statistical analysis was performed using R (version 3.5.1; R Core Team; Vienna, Austria). Coefficient and odds ratio (OR) estimates with 95% confidence intervals were reported, and *p*-values < 0.05 were considered indicative of statistical significance.

## 3. Results

### 3.1. Study Sample

In total, 28,056 individuals (65,778 visits) were followed up. At baseline, the median O_3_ peak-season concentration was 107.50 μg/m^3^ (25th to 75th percentile, 93.70–123.27 μg/m^3^), and the O_3_ annual mean concentration was 82.75 μg/m^3^ (25th to 75th percentile, 75.03–90.49 μg/m^3^). The distributions of O_3_ concentration for different time windows are shown in Supplemental Table S1a,b. Table 1 shows the baseline characteristics of the participants by peak-season O_3_ concentration quartile. Disease characteristics varied according to the O_3_ concentration. Despite using different measurements of exposure to O_3_, Table S2 shows the semblable characteristics. Supplementary Figure S1 shows the surveyed locations in seven Chinese geographical zones; the sample covered most provincial administrative regions in mainland China, except for Tibet.

### 3.2. Association between O_3_ Exposure and mRS Score

**Figure 1:**
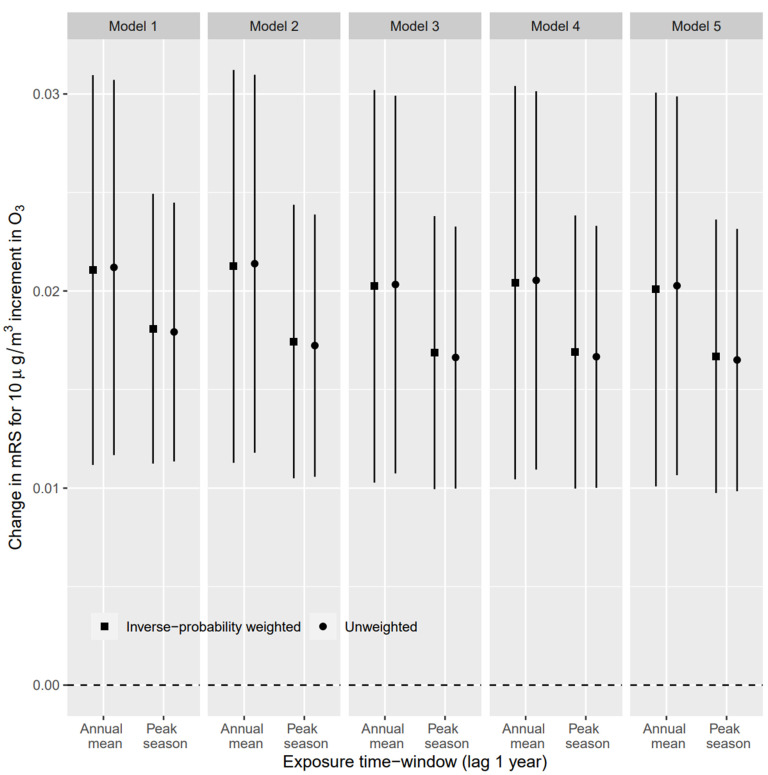
Associations of O_3_ exposure with mRS score based on different model settings. Model 1 was only adjusted for the interaction of the follow-up period with age at baseline and the interaction of the follow-up period with years after stroke at baseline. Model 2 was additionally adjusted for season. Model 3 was additionally adjusted for smoking, drinking, physical activity, milk intake, and body mass index. Model 4 was additionally adjusted for PM_2.5_. Model 5 was additionally adjusted for hypertension, diabetes, dyslipidemia, and atrial fibrillation. mRS, modified Rankin Scale.

**Figure 2:**
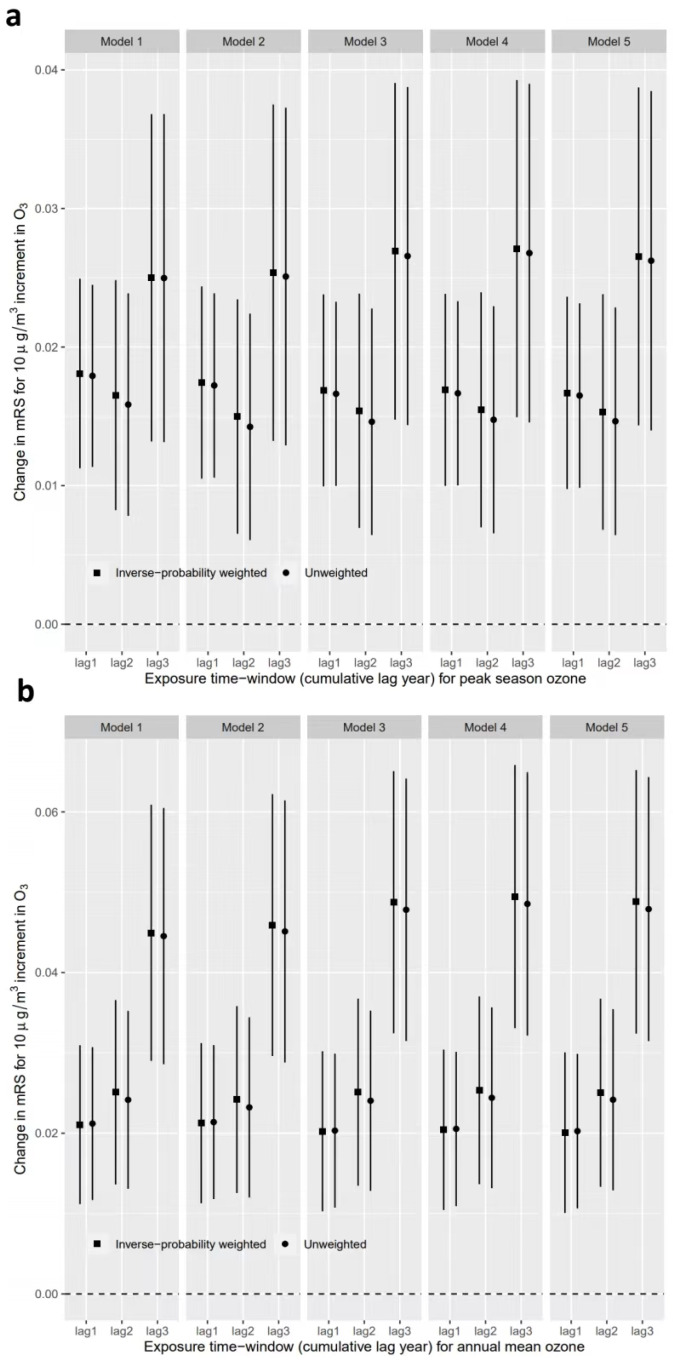
Associations of O_3_ exposure with mRS score using different exposure time windows. (**a**) Peak-season O_3_. (**b**) Annual mean O_3_. Models were adjusted for the interaction of the follow-up period with age at baseline, the interaction of follow-up period with years after stroke at baseline, season of the study, smoking, drinking, physical activity, milk intake, body mass index, hypertension, diabetes, dyslipidemia, and atrial fibrillation. mRS, modified Rankin Scale.

As shown in Figure 1, the effect of peak-season O_3_ was slightly weaker than that of the annual mean O_3_ in each model. Each 10 µg/m^3^ increment of annual average O_3_ exposure increased the mRS score by 0.020 points (95% CI, 0.010–0.030) in the fully adjusted model. The effect was estimated to be 0.017 (95% CI, 0.010–0.024) in terms of peak-season O_3_. The result was robust when adjusting for different covariates. For the secondary outcomes, there was a significant association between O_3_ exposure and an increase in mRS score. For each 10 µg/m^3^ increment in peak-season and annual mean O_3_ exposure, the OR of an increased mRS score (vs. unchanged or decreased mRS score) for peak-season and annual mean O_3_ was 1.23 (95% CI, 1.09–1.37) and 1.28 (95% CI, 1.09–1.52), respectively, in the fully adjusted model (Supplemental Figure S2). However, there was no significant effect on individuals with high levels of baseline mRS score (mRS > 2 points). In the sensitivity analyses, effect estimates of air pollutants back-extrapolated to the year of baseline examination were similar to or slightly higher than the main results (Figure 2).

### 3.3. Effect Modification

**Figure 3:**
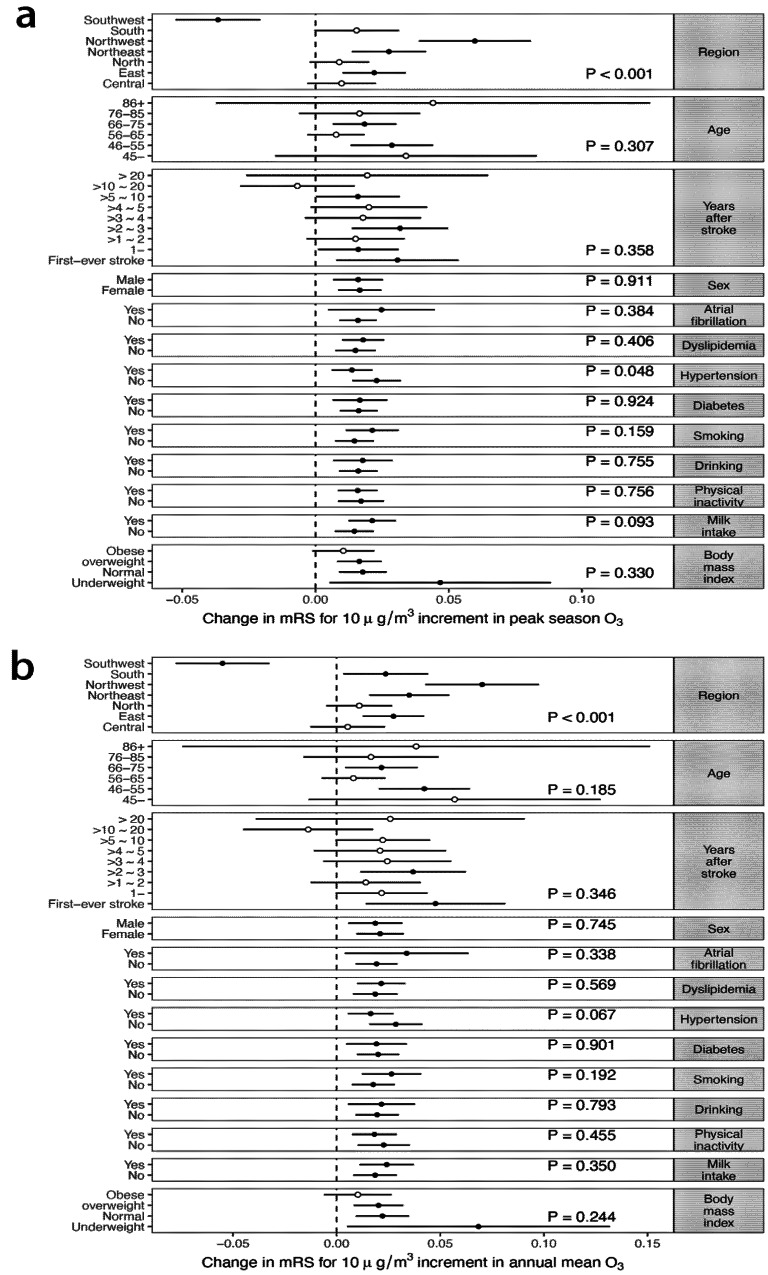
Subpopulation-specific effect of O_3_ exposure with mRS score outcomes for per 10 ug/m^3^ increment of O_3_ by demographic subgroups. (**a**) Peak-season O_3_. (**b**) Annual mean O_3_. Models were adjusted for the interaction of the follow-up period with age at baseline, the interaction of the follow-up period with years after stroke at baseline, season of the study, smoking, drinking, physical activity, milk intake, body mass index, hypertension, diabetes, dyslipidemia, and atrial fibrillation. mRS, modified Rankin Scale.

To identify the populations particularly susceptible to O_3_ exposure, we investigated the potential effect modifications of baseline characteristics (Figure 3). There was no change in effect stratified by age, years after stroke, sex, hypertension, diabetes, dyslipidemia, atrial fibrillation, drinking, smoking, physical inactivity, or BMI. The results showed significantly positive associations in northwest, northeast, and east China, with estimated effects of 0.060 (95% CI 0.039–0.081), 0.028 (95% CI 0.014–0.041), and 0.022 (95% CI 0.010–0.034), respectively, but not for southwest China (−0.037; 95%CI −0.053–−0.021). The difference across regions was statistically significant (*p* < 0.001). The relatively small sample size in southwest China (*n* = 4221, compared with *n* = 14,891 in north China) is likely the underlying reason; other possibilities are geographical differences in climate, lifestyle, residential habit of heating, and baseline health status. For instance, southwest had a high prevalence of stroke [15], which suggests a poor baseline level of cerebrovascular health. Therefore, among the vulnerable stroke patients, exposure to air pollution may lead to a fatal outcome rather than neurological disability. Further studies are warranted to confirm the modification effect and to examine the biological mechanisms of the geographical difference in the effect of O_3_ on disability after stroke.

### 3.4. Exposure−Response Relationship

**Figure 4:**
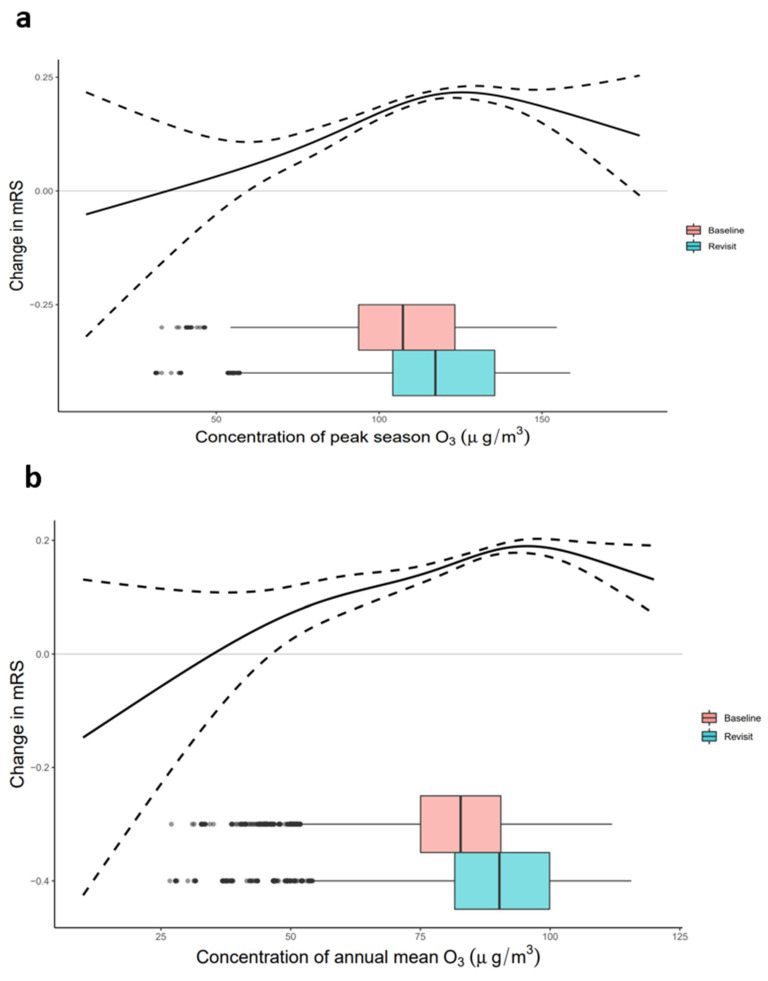
Exposure−response curves for O_3_ exposure levels with mRS score. The solid line represents the point estimates; the dashed line represents the confidence intervals. The distribution of O_3_ concentration of the baseline and revisit population are shown in the box plot. (**a**) Peak-season O_3_. (**b**) Annual mean O_3_. Models were adjusted for the interaction of the follow-up period with age at baseline, the interaction of the follow-up period with years after stroke at baseline, season of the study, smoking, drinking, physical activity, milk intake, body mass index, hypertension, diabetes, dyslipidemia, and atrial fibrillation. mRS, modified Rankin Scale.

When using splines for the annual mean O_3_ term in the nonlinear model, the exposure–response curve was similar to the curve using peak-season O_3_ exposure (Figure 4). The directions of the overall effects estimated by the nonlinear models were consistent with the linear results. The effect was almost linear at concentrations of ≤100 µg/m^3^ for peak-season O_3_. The estimated curvatures showed a saturated effect of O_3_ on post-stroke disability at high concentrations.

## 4. Discussion

China bears the highest burden of stroke globally [23]. However, few studies have evaluated the influence of the outdoor environment on stroke prognosis [24]. To the best of our knowledge, this is the only national longitudinal study to date to investigate the influence of O_3_ exposure on mRS score in China. We found that long-term exposure to O_3_ was associated with an elevated risk of neurological disability after controlling for potential confounders. Each 10 µg/m^3^ increment in peak-season O_3_ was associated with a 0.017 (95% CI 0.010–0.024) increment in the mRS score. The estimate was robust in various subpopulations. The exposure–response curve showed a sublinear function between the increased risk for post-stroke disability and an increased O_3_ concentration.

The risks of stroke mortality and hospital admission are associated with acute effects of O_3_ in China. A meta-analysis showed a weak association between short-term O_3_ and admission to hospital for stroke or mortality from stroke (relative risk, 1.001; 95% CI 1.000–1.002) per 10 ppb [9]. Previous reports on long-term O_3_ exposure and incidence of stroke are inconsistent. A study in the southeast United States revealed that the annual mean O_3_ was associated with an increased risk of stroke mortality with a hazard ratio (HR) of 1.012 (95% CI 1.012–1.013) [5]. Zhao et al. [8] reported an HR of 1.08 (95% CI 1.06–1.10) for each interquartile range increase in O_3_ (10.1 ppb) in a Canadian cohort. However, another study reported that warm-season O_3_ was not associated with an increase in the incidence of stroke (HR 0.96; 95% CI 0.91–1.01) [25]. The possible reasons for the discrepancy include the different study designs, exposure concentrations, and populations. Few studies have reported an association between exposure to O_3_ and an increased risk for disability. A cross-sectional study conducted in Texas from 2000 to 2012 reported that each 10 μg/m^3^ increment in daily exposure to O_3_ was significantly associated with a 0.29 (95% CI 0.06–0.51) increment of initial stroke severity (NIHSS).

Our results indicated that the effect of peak-season O_3_ was more concise than that of annual mean O_3_ with a narrower confidence interval. In some studies, O_3_ concentrations were calculated for the “peak” or warm season, whereas others used the annual average or median as an exposure indicator. The WHO has updated the Air Quality Guidelines to provide guidance on the health risks of O_3_. According to a meta-analysis concerning the long-term effect of O_3_ on mortality, the pooled OR from studies using annual mean metrics showed estimated effects for all-cause and respiratory mortality of 0.97 (95% CI 0.93–1.02) and 0.99 (95% CI 0.89–1.11) for each 10 μg/m^3^ increment of O_3_, respectively. In studies using peak-season O_3_ metrics, the pooled OR was estimated to be 1.01 (95% CI 1.00–1.02) for all-cause mortality and 1.02 (95% CI 0.99–1.05) for respiratory mortality. Although the meta-analysis identified high levels of heterogeneity, the error of the estimated effects measured using peak-season O_3_ was lower than annual mean O_3_, which is consistent with our findings. Therefore, peak-season mean may be useful for assessing the health effect of long-term O_3_ exposure.

Although the biological mechanisms underlying the adverse effects of O_3_ on the central nervous system remain unclear, some studies have implicated a few plausible pathways, including inflammation and oxidative stress. First, as an oxidizer, O_3_ can activate respiratory inflammatory responses directly, and cause systemic inflammations after entering the bloodstream [26,27]. Second, exposure to O_3_ can also induce endothelial dysfunction and increase the risk of thrombosis [28,29]. Third, because of oxidative stress, O_3_ exposure can introduce the formation of free radicals in cells and tissues, which furthers DNA damage and lipid peroxidation [30,31]. Finally, O_3_ has been found to change the permeability of the blood–brain barrier (BBB) and even to cause dysfunctions, which may further lead to persistent damage in the central nervous system [31,32].

This study had several limitations. First, in terms of exposure matching, O_3_ concentrations were estimated at a resolution of 10 km × 10 km. The geographic addresses were confined to the community-hospital level for confidentiality, possibly leading to exposure misclassification due to measurement error. Moreover, because stroke survivors spend more time indoors and we could not take into account indoor O_3_ concentration due to the inaccessibility of data, further research with a portable individual-level O_3_ exposure testing instrument is expected to validate our results. Second, except for PM_2.5_, we could not estimate the potential confounding or interactive effects of other ambient air pollutants such as NO_2_ and black carbon, which might be correlated with O_3_. After adjusting for PM_2.5_ as a covariate, the effect remained robust. Therefore, we assumed that the effect of O_3_ was independent of PM_2.5_. Third, the individual-level information was self-reporting, possibly introducing recall bias. Moreover, unmeasured confounders (e.g., medication history) might bias the results in an unknown direction. Fourth, the unbalanced age structure of participants (≥40 years) and sampled counties (more concentrated in the eastern part of the country) make this study less representative of the whole population. The participants that were excluded due to having no follow-up measurements might also lead to selection bias. In addition, people with a higher baseline mRS score may have a higher risk for death and thus be less likely to be followed up. In the sensitivity analysis, the results obtained with and without weighting were similar, demonstrating the robustness of the findings. Fifth, the mRS score might be influenced by stroke severity and recurrence, and the precise times of stroke or transient ischemic attack (also known as mini stroke) are unknown, which might have biased the results. However, we stratified the studied population by years after stroke in the sensitivity analysis, but did not find a significant difference between the subpopulation-specific associations. The results of the sensitivity analysis indicated that the history of stroke did not considerably affect our results.

## 5. Conclusions

Long-term exposure to O_3_ was associated with a higher risk for post-stroke neurological disability among middle-aged and elderly Chinese people. Taking ambient O_3_ under control might delay the progression of neurological disability among the aging population. Our findings point to the urgent necessity for implementing stringent clean air policies to reduce ambient O_3_ pollution, which might bring great public health benefits in an aging society.

## Figures and Tables

**Table 1:** Descriptive characteristics of the study participants at baseline by peak-season O_3_ concentration quartile.

	Overall	O_3_ First Quartile	O_3_ Second Quartile	O_3_ Third Quartile	O_3_ Fourth Quartile	*p*-Value
		(≤93.70 μg/m^3^)	(93.70–107.50 μg/m^3^)	(107.50–123.27 μg/m^3^)	(>123.27 μg/m^3^)	
**Age Group**						<0.01
≤45	585 (2.09)	197 (2.49)	161 (2.29)	125 (1.78)	102 (1.46)	——
45–55	4288 (15.28)	1176 (14.84)	1016 (14.48)	1066 (15.15)	1030 (14.76)	——
55–65	9857 (35.13)	2438 (30.77)	2405 (34.27)	2486 (35.33)	2528 (36.22)	——
65–75	9681 (34.51)	2308 (29.13)	2447 (34.87)	2437 (34.64)	2489 (35.66)	——
75–85	3388 (12.08)	829 (10.46)	912 (13.00)	865 (12.29)	782 (11.21)	——
>85	257 (0.92)	76 (0.96)	76 (1.08)	57 (0.81)	48 (0.69)	——
**Sex**						<0.01
Female	13,094 (46.67)	3398 (48.38)	3353 (47.78)	3267 (46.43)	3076 (44.08)	——
Male	14,842 (52.90)	3610 (51.40)	3659 (52.14)	3713 (52.77)	3860 (55.31)	——
Missing	120 (0.43)	16 (0.23)	5 (0.07)	56 (0.80)	43 (0.62)	——
**Atrial Fibrillation**						<0.01
No	26,649 (94.99)	6661 (94.83)	6537 (93.16)	6736 (95.74)	6715 (96.22)	——
Yes	1401 (4.99)	363 (5.17)	480 (6.84)	294 (4.18)	264 (3.78)	——
Missing	6 (0.02)	0 (0.00)	0 (0.00)	6 (0.09)	0 (0.00)	——
**Dyslipidemia**						<0.01
No	15,179 (54.1)	3484 (49.6)	3959 (56.42)	4068 (57.82)	3668 (52.56)	——
Yes	9715 (34.63)	1902 (27.08)	2242 (31.95)	2609 (37.08)	2962 (42.44)	——
Missing	3162 (11.27)	1638 (23.32)	816 (11.63)	359 (5.10)	349 (5.00)	——
**Hypertension**						<0.01
No	9021 (32.15)	2493 (35.49)	2174 (30.98)	2249 (31.96)	2105 (30.16)	——
Yes	19,029 (67.83)	4531 (64.51)	4843 (69.02)	4781 (67.95)	4874 (69.84)	——
Missing	6 (0.02)	0 (0.00)	0 (0.00)	6 (0.09)	0 (0.00)	——
**Diabetes Mellitus**						<0.01
No	20,847 (74.30)	4908 (69.87)	5289 (75.37)	5383 (76.51)	5267 (75.47)	——
Yes	5272 (18.79)	1027 (14.62)	1278 (18.21)	1484 (21.09)	1483 (21.25)	——
Missing	1937 (6.90)	1089 (15.50)	450 (6.41)	169 (2.40)	229 (3.28)	——
**Smoke**						<0.01
No	18,217 (64.93)	4154 (59.14)	4626 (65.93)	4796 (68.16)	4641 (66.50)	——
Yes	7133 (25.42)	1779 (25.33)	1824 (25.99)	1793 (25.48)	1737 (24.89)	——
Missing	2706 (9.64)	1091 (15.53)	567 (8.08)	447 (6.35)	601 (8.61)	——
**Drink**						<0.01
No	23,133 (82.45)	5812 (82.74)	5862 (83.54)	5845 (83.07)	5614 (80.44)	——
Yes	4910 (17.50)	1210 (17.23)	1152 (16.42)	1184 (16.83)	1364 (19.54)	——
Missing	13 (0.05)	2 (0.03)	3 (0.04)	7 (0.10)	1 (0.01)	——
**Sport**						<0.01
No	11,147 (39.73)	2824 (40.21)	2673 (38.09)	2660 (37.81)	2990 (42.84)	——
Yes	16,901 (60.24)	4198 (59.77)	4344 (61.91)	4370 (62.11)	3989 (57.16)	——
Missing	8 (0.03)	2 (0.03)	0 (0.00)	6 (0.09)	0 (0.00)	——
**Milk**						<0.01
No	17,073 (60.85)	3837 (54.63)	4264 (60.77)	4304 (61.17)	4668 (66.89)	——
Yes	4449 (15.86)	973 (13.85)	1130 (16.10)	1090 (15.49)	1256 (18.00)	——
Missing	6534 (23.29)	2214 (31.52)	1623 (23.13)	1642 (23.34)	1055 (15.12)	——
**BMI**						<0.01
(−Inf,18.5]	545 (1.94)	163 (2.32)	175 (2.49)	124 (1.76)	83 (1.19)	——
(18.5,24]	10,580 (37.71)	3092 (44.02)	2850 (40.62)	2435 (34.61)	2203 (31.57)	——
(24,28]	11,849 (42.23)	2786 (39.66)	2906 (41.41)	3105 (44.13)	3052 (43.73)	——
(28, Inf]	5055 (18.02)	967 (13.77)	1082 (15.42)	1370 (19.47)	1636 (23.44)	——
Missing	27 (0.10)	16 (0.23)	4 (0.06)	2 (0.03)	5 (0.07)	——

## Data Availability

Additional data are not available, because only authorized researchers can assess the database.

## Supplementary Materials

The following are available online at https://www.mdpi.com/article/10.3390/biology11081216/s1, Table S1. (a) Distribution of different quantiles of O_3_ peak season concentration for lag 1, 2, 3 year (μg/m^3^); (b) Distribution of different quantiles of O_3_ annual mean concentration for lag 1, 2, 3 year (μg/m^3^). Table S2. Descriptive characteristics of study participants at baseline by annual averaged O_3_ concentration quartile. Figure S1. Locations of surveyed counties of CNSSS from 2014 to 2019. (+) represents the locations of the surveyed counties. Figure S2. Associations of O_3_ exposure with different categorical mRS score outcomes for per 10 μg/m^3^ increment of O_3_. a: Peak season O_3_. b: Annual mean O_3_. Model 1 was only adjusted for the interaction of follow-up period with age at baseline, and the interaction of follow-up period with years after stroke at baseline. Model 2 was additionally adjusted for season. Model 3 was additionally adjusted for smoking, drinking, physical activity, milk intake, and body mass index. Model 4 was additionally adjusted for PM2.5. Model 5 was additionally adjusted for hypertension, diabetes, dyslipidemia and atrial fibrillation. mRS, modified Rankin Scale.
