# Supplementary material for: Long-Term Exposure to Ozone Increases Neurological Disability after Stroke: Findings from a Nationwide Longitudinal Study in China
Source: Biology (Basel). 2022 Aug 13;11(8):1216. doi: 10.3390/biology11081216 (PMC9404899; doi:10.3390/biology11081216)
Supplement: Supplementary file 1 [file biology-11-01216-s001.zip › biology-1775967-supplementary.pdf]

Supplemental Materials

Long-term exposure to ozone increases neurological disability after stroke: findings from a nationwide longitudinal study in China

Jiajianghui Li [1,†], Hong Lu [1,†], Man Cao [2], Mingkun Tong [1], Ruohan Wang [1], Xinyue Yang [3], Hengyi Liu [1], Qingyang Xiao [4], Baohua Chao [5], Yuanli Liu [2], Tao Xue [1,*] and Tianjia Guan [2,*]

1 Institute of Reproductive and Child Health/National Health Commission Key Laboratory of Reproductive Health and Department of Epidemiology and Biostatistics, School of Public Health, Peking University,
Beijing, 100191 China; lijiajianghuizi@163.com (J.L.); luhongsph@hsc.pku.edu.cn (H.L.);
tongmk2021@bjmu.edu.cn (M.T.); wangruohan1202@163.com (R.W.); 1610306242@bjmu.edu.cn (H.L.)

2 School of Health Policy and Management, Chinese Academy of Medical Sciences & Peking Union Medical College, Beijing, 100730 China; caomanpumc@163.com (M.C.); yliu_pumc@163.com (Y.L.)

3 College of Environmental Sciences and Engineering, Peking University, Beijing, 100871 China; 2101213369@stu.pku.edu.cn

4 State Key Joint Laboratory of Environmental Simulation and Pollution Control, School of Environment, Tsinghua University, Beijing 100084, China; qingyang_xiao18@163.com

5 The General Office of Stroke Prevention Project Committee, National Health Commission of the People's Republic of China, Beijing, 100053 China; chaobh@163.com

* Correspondence: xuetaogk_9032@126.com (T.X.); gtj@sph.pumc.edu.cn (T.G.)

† These authors contributed equally to this work.

Supplemental Table S1 **(a)** Distribution of different quantiles of O_3 peak season concentration for lag 1,2,3 year（μg/m^3） (**b**) Distribution of different quantiles of O_3 annual mean concentration for lag 1,2,3 year（μg/m^3）

(a)

	10%	25%	50%	75%	90%	min	max	mean	n	visit
lag1O_3	86.70	99.07	112.57	127.68	140.63	31.41	158.71	113.18	28056	65778
lag2O_3	85.77	97.19	110.19	124.80	136.78	33.23	154.26	110.57	26084	58245
lag3O_3	84.35	95.30	108.36	121.21	128.65	38.81	154.57	107.55	19229	39751

(**b**)

	10%	25%	50%	75%	90%	min	max	mean	n	visit
lag1O_3	70.40	78.35	87.46	95.22	104.23	26.72	115.63	86.49	28056	65778
lag2O_3	68.17	77.21	84.58	92.63	100.60	26.58	112.40	84.32	26084	58245
lag3O_3	68.05	75.88	82.60	90.63	95.04	31.84	109.75	82.05	19229	39751

Supplemental Table S2 Descriptive characteristics of study participants at baseline by annual averaged O_3 concentration quartile

	Overall	O_3 First quartile (≤75.03μg/m^3)	O_3 Second quartile (75.03-82.75μg/m^3)	O_3 Third quartile (82.75-90.49μg/m^3)	O_3 Fourth quartile (>90.49μg/m^3)	p-Value
Age Group						<0.01
≤45	585(2.09)	201(2.87)	137(1.95)	136(1.94)	111(0.16)	——
45-55	4288(15.28)	1154(16.45)	1043(14.86)	1060(15.12)	1031(1.47)	——
55-65	9857(35.13)	2408(34.33)	2539(36.18)	2414(34.43)	2496(3.56)	——
65-75	9681(34.51)	2349(33.49)	2338(33.31)	2489(35.50)	2505(3.57)	——
75-85	3388(12.08)	831(11.85)	886(12.62)	858(12.24)	813(1.16)	——
>85	257(0.92)	72(1.03)	75(1.07)	54(0.77)	56(0.08)	——
Sex						<0.01
Female	13094(46.67)	3337(47.57)	3419(48.72)	3138(44.76)	3200(45.64)	——
Male	14842(52.90)	3670(52.32)	3534(50.36)	3826(54.57)	3812(54.36)	——
Missing	120(0.43)	8(0.11)	65(0.93)	47(0.67)	0(0.00)	——
AF						<0.01
No	26649(94.99)	6688(95.34)	6633(94.51)	6542(93.31)	6786(96.78)	——
Yes	1401(4.99)	327(4.66)	385(5.49)	463(6.6)	226(3.22)	——
Missing	6(0.02)	0(0.00)	0(0.00)	6(0.09)	0(0.00)	——
Dyslipidemia						<0.01
No	15179(54.1)	3381(48.2)	4043(57.61)	3868(55.17)	3887(55.43)	——
Yes	9715(34.63)	2003(28.55)	2453(34.95)	2459(35.07)	2800(39.93)	——
Missing	3162(11.27)	1631(23.25)	522(7.44)	684(9.76)	325(4.63)	——
Hypertension						<0.01
No	9021(32.15)	2457(35.02)	2198(31.32)	2236(31.89)	2130(30.38)	——
Yes	19029(67.83)	4558(64.98)	4820(68.68)	4769(68.02)	4882(69.62)	——
Missing	6(0.02)	0(0.00)	0(0.00)	6(0.09)	0(0.00)	——
Diabetes Mellitus						<0.01
No	20847(74.3)	4692(66.89)	5523(78.7)	5234(74.65)	5398(76.98)	——
Yes	5272(18.79)	1163(16.58)	1331(18.97)	1359(19.38)	1419(20.24)	——

Missing	1937(6.9)	1160(16.54)	164(2.34)	418(5.96)	195(2.78)	——
Smoke						<0.01
No	18217(64.93)	4083(58.2)	4749(67.67)	4759(67.88)	4626(65.97)	——
Yes	7133(25.42)	1732(24.69)	1888(26.9)	1667(23.78)	1846(26.33)	——
Missing	2706(9.64)	1200(17.11)	381(5.43)	585(8.34)	540(7.7)	——
Drink						<0.01
No	23133(82.45)	5854(83.45)	5795(82.57)	5816(82.96)	5668(80.83)	——
Yes	4910(17.5)	1156(16.48)	1223(17.43)	1188(16.94)	1343(19.15)	——
Missing	13(0.05)	5(0.07)	0(0.00)	7(0.1)	1(0.01)	——
Sport						<0.01
No	11147(39.73)	2845(40.56)	2670(38.05)	2788(39.77)	2844(40.56)	——
Yes	16901(60.24)	4168(59.42)	4348(61.95)	4217(60.15)	4168(59.44)	——
Missing	8(0.03)	2(0.03)	0(0.00)	6(0.09)	0(0.00)	——
Milk						<0.01
No	17073(60.85)	3604(51.38)	4330(61.7)	4288(61.16)	4851(69.18)	——
Yes	4449(15.86)	1153(16.44)	1094(15.59)	1164(16.6)	1038(14.8)	——
Missing	6534(23.29)	2258(32.19)	1594(22.71)	1559(22.24)	1123(16.02)	——
BMI						<0.01
(-Inf,18.5]	545(1.94)	150(2.14)	150(2.14)	151(2.15)	94(1.34)	——
(18.5,24]	10580(37.71)	2964(42.25)	2748(39.16)	2591(36.96)	2277(32.47)	——
(24,28]	11849(42.23)	2875(40.98)	2916(41.55)	2997(42.75)	3061(43.65)	——
(28, Inf]	5055(18.02)	1008(14.37)	1203(17.14)	1270(18.11)	1574(22.45)	——
Missing	18(0.26)	1(0.01)	2(0.03)	6(0.09)	0(0.00)	——

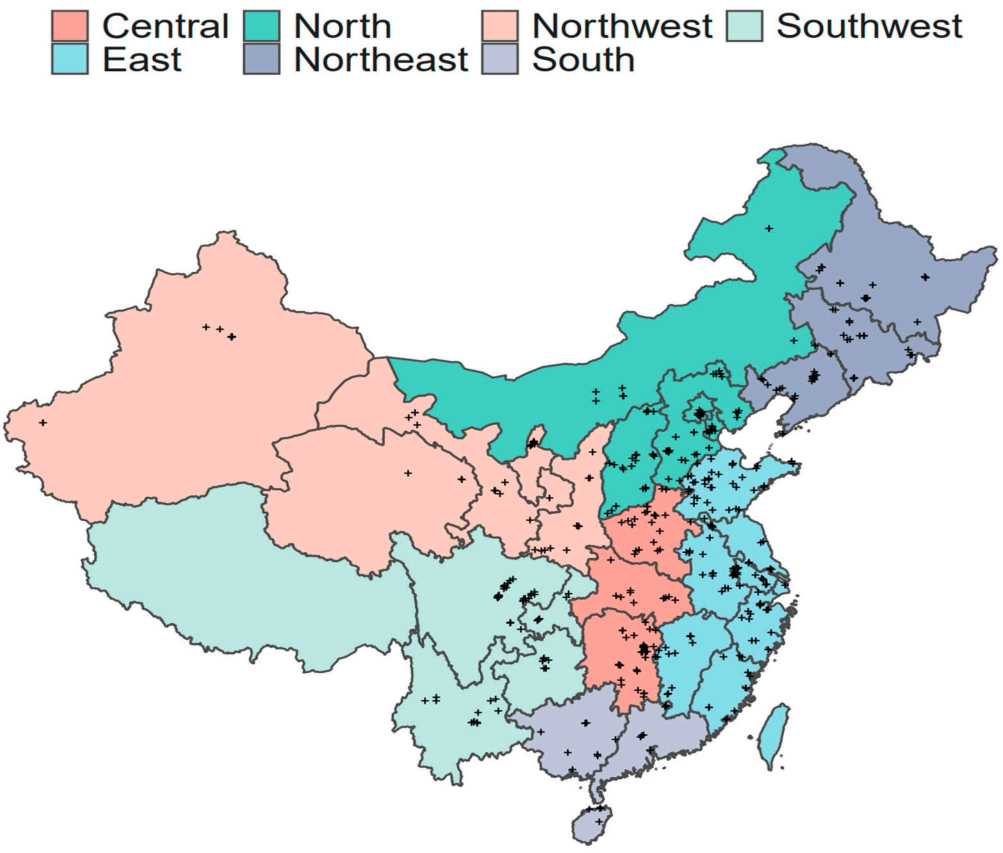

Supplementary Figure S1. Locations of surveyed counties of CNSSS from 2014 to 2019. (+) represents the locations of the surveyed counties.

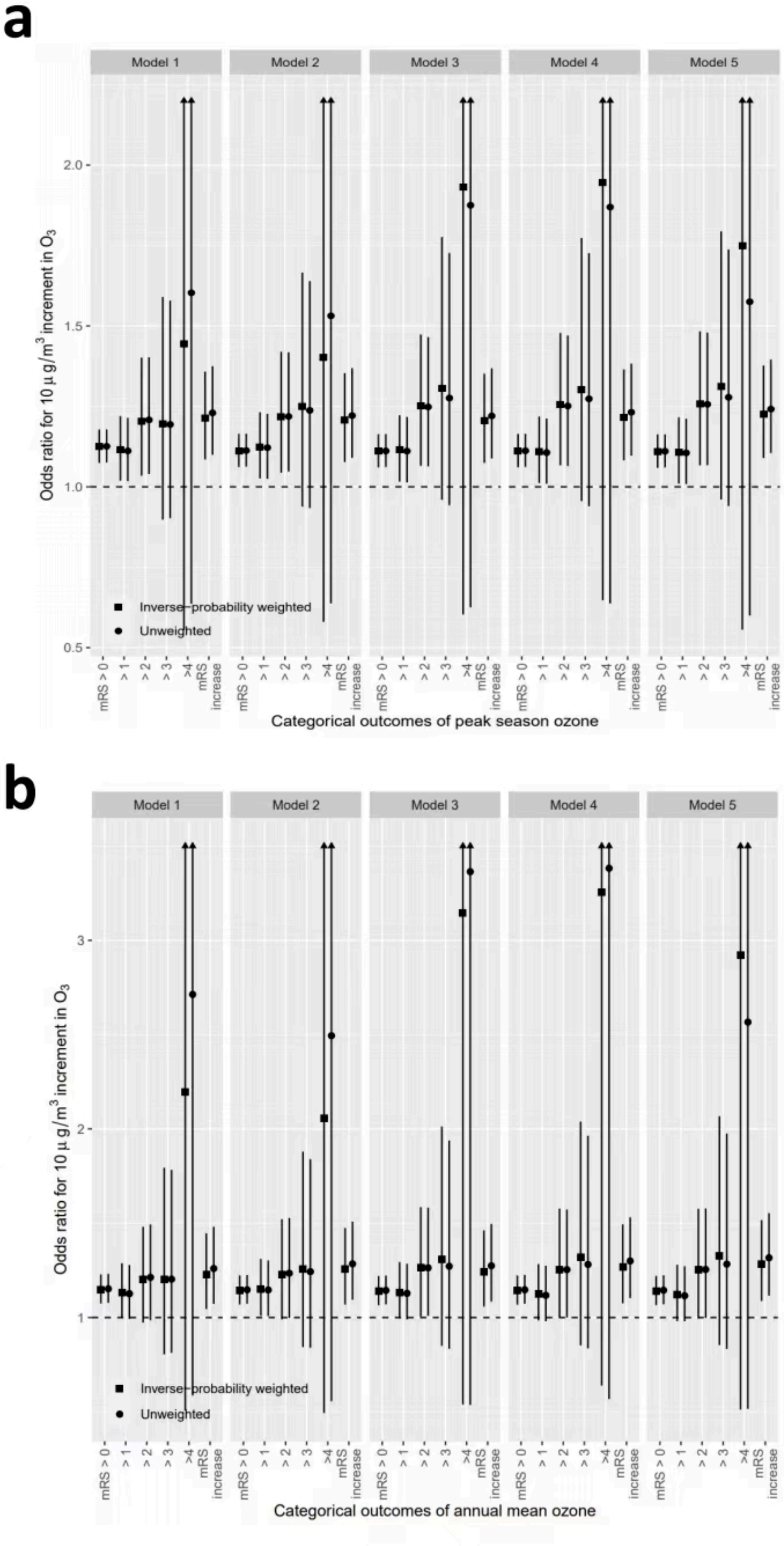

Supplemental Figure S2. Associations of O_3 exposure with different categorical mRS score outcomes for per 10 ug/m^3 increment of O_3. a: Peak season O_3. b: Annual mean O_3. Model 1 was only adjusted for the interaction of follow-up period with age at baseline, and the interaction of follow-up period with years after stroke at baseline. Model 2 was additionally adjusted for season. Model 3 was additionally adjusted for smoking, drinking, physical activity, milk intake, and body mass index. Model 4 was additionally adjusted for $PM_{2.5}$.

Model 5 was additionally adjusted for hypertension, diabetes, dyslipidemia and atrial fibrillation. mRS, modified Rankin Scale.
